# Determination of the toxicity of the freshwater cyanobacterium *Woronichinia naegeliana* (Unger) Elenkin

**DOI:** 10.1007/s10811-017-1062-1

**Published:** 2017-02-03

**Authors:** Beata Bober, Jan Bialczyk

**Affiliations:** Department of Plant Physiology and Development, Faculty of Biochemistry, Biophysics and Biotechnology, Jagiellonian University, Gronostajowa 7, 30-387 Krakow, Poland

**Keywords:** Bioassays, Cyanobacteria, Cyanopeptides, Microginins

## Abstract

Cyanobacterial blooms are undesirable for ecological and health reasons. While *Woronichinia naegeliana* is a cyanobacterial species that appears frequently in freshwater, information about it is limited. An evaluation of its toxicity was conducted via tests based on the crustaceans *Thamnocephalus platyurus* and *Daphnia pulex*. The greatest effect of the aqueous extract obtained from *W. naegeliana* cells was observed for *T. platyurus*. The denoted semi-lethal concentration (LC_50_) after 24 h of exposure was 0.99 mg of dry weight (d.w.) mL^−1^. A lower toxicity was displayed for *D. pulex*, although it grew with time. Among the 18 fractions separated from cyanobacterial extract, only one containing the microginin FR3 (MG-FR3) displayed biological activeness against *T. platyurus*. The remaining products synthesized by *W. naegeliana* displayed an absence or a low level of toxicity making it impossible to determine the LC_50_ value. Detailed research revealed that MG-FR3 did not affect the activity of enzymes such as trypsin, chymotrypsin, elastase and thrombin, which indicates another mode of action. The results demonstrated that blooms of *W. naegeliana* showed toxic activity towards invertebrate zooplankton.

## Introduction

The intensification of cyanobacterial mass occurrences in various aquatic environments is observed worldwide. Cyanobacterial blooms are serious problems, particularly for reservoirs that are a source for potable water. Cyanobacteria are known for the production of a wide range of secondary metabolites of various structure and biological activity. Among them are compounds affecting the taste and odour of water (e.g. 2-methylisoborneol [MIB] and geosmin), toxins (e.g. alkaloids, cyclic peptides) and oligopeptides, whose biological function is unclear (see references in Smith et al. 2008). Although cyanobacteria are capable of simultaneous synthesis of several compounds, only a few are dominant. Generally, cyanobacteria are recognized as toxic if they possess the ability to produce neurotoxins (e.g. anatoxin-a), dermatotoxins (e.g. aplysiatoxin) or hepatotoxins (e.g. microcystins) (Burkholder and Gilbert 2006). This classification does not concern the synthesis of some oligopeptides that contain unusual amino acids in their structures and are considered ‘non-toxic’, although some of them have been recognized as protease inhibitors. The strong inhibitory activity of cyanobacterial peptides against animal digestive proteases (e.g. elastase, chymotrypsin, trypsin) might indicate their function as anti-grazing factors, whereas the inhibition of thrombin activity, final coagulation protease, might be useful from a medical point of view in designing of new drugs (see references in Chlipala et al. 2011).

The monitoring of potential threats caused by cyanobacterial blooms relies on confirmation of the presence of toxins using mostly analytical methods. However, the application of chemical methods is often complicated due to the lack of appropriate equipment, operational procedures or commercially available standards for compounds. In addition, based only on the results obtained from chemical analyses, it is difficult to exclude the synergic effects of several toxic and non-toxic compounds released by cyanobacteria. Jungmann (1992) showed that fractions isolated from the extract of *Microcystis aeruginosa* cells that did not contain hepatotoxic microcystin-LR appeared to be more toxic to *Daphnia* sp. than those containing the compound. These may be the most probable reasons why an application of bioassays for evaluation of the bioactivity of all secondary metabolites synthesized by cyanobacteria increases in time. Additionally, relatively simple procedures keep the costs of these routinely performed analyses relatively low (Törökné et al. 2007).

Out of all the cyanobacterial species commonly occurring in water reservoirs, the most widely characterized are those frequently described as toxic (Sivonen and Jones 1999). Although blooms of the cyanobacterium *Woronichinia naegeliana* belonging to the *Chroococcales* also appear more frequently in the phytoplankton of freshwater worldwide; information about this species is limited. This is likely due to difficulties in the cultivation of this species under laboratory conditions (Rajaniemi-Wacklin et al. 2005). Bober et al. (2011) reported determination of compounds produced by *W. naegeliana*. The majority of its identified secondary metabolites belong to three classes of oligopeptides: microginins, cyanopeptolins and anabaenopeptins. The extract also contained trace amounts of microcystin-LR. Some studies of *W. naegeliana* report contradictory data about its toxicity (Rajaniemi–Wacklin et al. 2005, Willame et al. 2005, Baudin et al. 2006, Oberholster et al. 2006). However, in these studies, the main criteria for cyanobacterial biological activity were the presence of microcystins or genes responsible for its production.

Due to potentially significant consequences following the occurrence of *W. naegeliana* blooms in freshwater and the limited information about its biological activity, this study has been undertaken to determine its toxicity. The toxicity tests used here were based on the crustaceans *Thamnocephalus platyurus* and *Daphnia pulex*. These assays are commonly used to evaluate the toxicity of many compounds and are particularly sensitive to cyanobacterial toxins. In addition, assays on separated fractions isolated from cyanobacterial cell extract were performed to determine which compounds are responsible for the biological response. More detailed research including various protease inhibition assays was conducted on the microginin FR3, the dominant compound synthesized by *W. naegeliana*.

## Material and methods

### Cyanobacteria

*Woronichinia naegeliana* (Unger) Elenkin was collected from Dobczyce reservoir in southern Poland in September 2013. Identification and isolation were done according to Bober et al. (2011).

### Sample preparation

The cell material was lyophilized and divided into two portions of 1 g dry weight (d.w.) each. One portion was extracted with 100 mL Milli-Q water and then filtered through GF/C glass-fibre filters (Whatman, UK). The second portion was used for separation of crude cell extract into fractions containing secondary metabolites according to the procedure described previously (Bober et al. 2011). Briefly, lyophilized cells were extracted with 100 mL 100% methanol under constant shaking and then filtered through a GF/C filter. After evaporation to dryness in a nitrogen atmosphere at room temperature, the sample was dissolved in 100 mL Milli-Q water and concentrated by solid phase extraction with a C_18_ silica cartridge (Baker Bond, USA). The cartridge was conditioned with 10 mL 100% methanol followed by 10 mL methanol: 10% acetic acid (4:1 *v*/*v*). The aqueous sample was passed through at 5 mL min^−1^ flow rate. The cartridge was then washed with 10 mL portions of 10, 20 and 30% methanol. The eluate, obtained using 10 mL 80% methanol, was evaporated to dryness in a nitrogen atmosphere at room temperature and dissolved in 1 mL Milli-Q water for analysis using a Waters Inc. (USA) high-performance liquid chromatography system containing a 600E gradient pump, 717 plus autosampler, a Jetstream 2 plus column thermostat, 996 photodiode array detector and Millenium^32^ SS Software with PDA option. Separation of extract was achieved using a Symmetry C_18_ column (4.6 × 250 mm; 5 μm; Waters, USA) maintained at 23 °C and gradient mobile phases consisting of (a) water/trifluoroacetic acid (0.05%, *v*/*v*) and (b) acetonitrile/trifluoroacetic acid (0.05%, *v*/*v*). The elution gradient was changed from 70 to 35% of eluent A over 35 min at a flow rate 0.7 mL min^−1^. Chromatograms were monitored at 220 nm. The fractions separated were evaporated to dryness in a nitrogen atmosphere at room temperature and diluted with Milli-Q water to the volume of the extract that was separated; therefore, their concentrations were equivalent to *W. naegelina* biomass concentration. The fractions were analysed using the ULPC-MS/MS system consisting of a Waters ACQUITY UPLC (Waters Inc., USA) coupled with a Waters TQD mass spectrometer (electrospray ionization mode ESI-tandem quadrupole). Chromatographic analyses were performed using the Acquity UPLC BEH (bridged ethyl hybrid) C_18_ column (2.1 × 100 mm; 1.7 μm) equipped with the Acquity UPLC BEH C_18_ VanGuard pre-column (2.1 × 5 mm; 1.7 μm). The column was maintained at 40 °C and eluted using mobile phases consisting of (a) water/formic acid (0.1%, *v*/*v*) and (b) acetonitrile/formic acid (0.1%, *v*/*v*). Gradient conditions changed from 95 to 0% of eluent A over 10 min at a flow rate of 0.3 mL min^−1^. The MS operational conditions were as follows: source temperature of 150 °C, desolvation temperature of 350 °C, desolvation gas flow rate of 600 L h^−1^, cone gas flow of 100 L h^−1^, capillary potential of 3.00 kV and cone potential of 20 V. Nitrogen was used for both the nebulizing and drying gas. The data were obtained in a scan mode ranging from 50 to 2000 m/z. Target ions selected for MS/MS analyses were fragmented with a collision-activated dissociation energy of 40 eV. The ion spectra were obtained by scanning from the 50 to 1100 m/z range. The MassLynx V 4.1 (Waters Inc., USA) software was used for data evaluation. The identities of the compounds presented in each fraction were determined according to Bober et al. (2011).

### Toxicity assays

The dilution series of the aqueous crude extract and separated fractions were prepared with appropriate exposure media in the range of 0.1 to 10 mg d.w. mL^−1^. The evaluation of cyanobacterial extract toxicity was performed through the application of commercially available acute toxicity assays based on the crustaceans *Thamnocephalus platyurus* (THAMNOTOXKIT F) and *Daphnia pulex* (DAPHNOTOXKIT F) following the respective standard operational procedures (Microbiotests Inc., Belgium). The quantitative importance of the toxic effects was calculated as a 50% effective concentration. Mortality (LC_50_) of *T. platyurus* larvae was recorded after exposure to the cyanobacterial extract for 24 h, whereas dead and immobile (EC_50_) *D. pulex* were estimated after 24 and 48 h. In both assays, appropriate exposure media were used as a control. The bioassays were considered valid if the mortality or immobilization of test organisms in the controls did not exceed 10%. The toxicity of separated fractions was performed only against *T. platyurus*.

### Enzyme inhibition assays

Detailed analysis of the toxicity mechanism used for the following enzymes, trypsin, chymotrypsin, elastase and thrombin, was performed only for the fraction, which gave a positive result in the crustaceans’ bioassays. This fraction was dissolved in ethanol and then diluted in appropriate buffers to perform the respective enzyme assays.

#### Trypsin inhibition assays

Inhibition assays of trypsin (EC 3.4.21.4) were performed according to the modified method of Reshef and Carmeli (2001). Trypsin was dissolved in 50 mM TRIS-HCl/100 mM NaCl/1 mM CaCl_2_ pH 7.5 to prepare 1 mg mL^−1^ solutions. This buffer was also used for the preparation of the 2 mM substrate solution of *N*-benzoyl-d,l-arginine-*p*-nitroanilide. The reaction mixture, which contained 100 μL of buffer, 10 μL of enzyme solution and 10 μL of test solution, was added to a microtiter plate well and pre-incubated at 37 °C for 5 min followed by the addition of 100 μL substrate solution. The absorbance of the solutions was measured immediately at 405 nm and after 30-min incubation at 37 °C.

#### Chymotrypsin inhibition assays

Inhibition assays of chymotrypsin (EC 3.4.21.1) were performed according to the method of Kisugi and Okino (2009). Briefly, chymotrypsin was dissolved in 50 mM TRIS-HCl pH 7.6 to prepare a 15 U mL^−1^ solution. This buffer was used for the preparation of the 1 mg mL^−1^ substrate solution of *N*-succinyl-l-phenylalanyl-*p*-nitroanilide. The reaction mixture, which contained 30 μL of buffer, 50 μL of enzyme solution and 10 μL of test solution, was added to a microtiter plate well and pre-incubated at 37 °C for 5 min, followed by the addition of 100 μL substrate solution. The absorbance of the solutions was measured immediately at 405 nm and after 30-min incubation at 37 °C.

#### Elastase inhibition assay

This inhibition assay was determined by modifying the method described by Grach-Pogrebinsky et al. (2003). The reaction mixture, which contained 150 μL of 0.2 M TRIS-HCl buffer (pH 8.0), 10 μL of elastase (EC 3.4.21.36) (0.0075 mg/mL in 0.2 M TRIS-HCl buffer (pH 8.0)) and 10 μL of test solution, was added to each microtiter plate well. Then, the mixture was pre-incubated at 30 °C for 20 min, and 30 μL of *N*-succinyl-Ala-Ala-Ala-*p*-nitroanilide (2 mM in 0.2 M TRIS-HCl buffer (pH 8.0)) was added. The absorbance of the solutions was read immediately at 405 nm and after incubation at 30 °C for 30 min.

#### Thrombin inhibition assay

Thrombin (EC 3.4.21.5) inhibitory activity was measured according to Anas (2012). Briefly, the reaction mixture, which contained 90 μL of thrombin from bovine plasma (1.3 U mL^−1^ in 0.15 M TRIS-imidazole (pH 8.2)) and 20 μL of test sample, was pre-incubated at 37 °C for 5 min. Then, 90 μL of Bz-Phe-Val-Arg-pNa.HCl (200 μg mL^−1^ in 0.2 M TRIS-HCl buffer (pH 8.0)) was added to begin the reaction. The absorbance was measured immediately and after incubation at 37 °C for 30 min.

### Chemicals

All reagents were analytical, HPLC or MS grade and were from Sigma-Aldrich (USA) or delivered within crustacean toxicity screening tests (Microbiotests Inc., Belgium). Ultrapure grade water (Milli-Q water) was obtained from Millipore (USA).

### Statistical analysis

Calculations of toxicity assays based on crustaceans were made using probit analysis (U.S. EPA 1985). Briefly, the percentages of toxic effects were plotted against log concentration, and the 50% effective concentrations were calculated using analysis of regression. Data obtained from enzyme assays were analysed using a sigmoidal dose-response curve and BioDataFit 1.02 software (Chang Bioscience Inc.). All data are expressed as the mean ± standard deviation (SD) of five replicates.

## Results

### Toxicity of *W. naegeliana* cell extract

Among the test organisms, the greatest biological sensitivity to the effect of the aqueous extract from *W. naegeliana* cells was displayed by *T. platyurus* (Table 1). The denoted lethal concentration on 50% of individuals after 24-h exposure to cyanobacterial extract was 0.99 mg d.w. mL^−1^. The effective concentration of 5.21 mg d.w. mL^−1^ caused death or immobilization of *D. pulex*. However, two times longer exposure to cyanobacterial extract (48 h) caused reduction of the effective concentration by 1.77 times.

**Table 1:** Toxicity of the extract from *Woronichinia naegeliana* cells assessed with crustaceans’ bioassays expressed as 50% effective concentrations (LC_50_—mortality concentration; EC_50_—mobility inhibitory concentration). Data are expressed as mean ± SD (*n* = 5)

Extract exposure			
Organism	Time (h)	Response type	Concentration (mg dry weight mL^−1^)
*Thamnocephalus platyurus*	24	LC_50_	0.99 ± 0.25
*Daphnia pulex*	24	EC_50_	5.21 ± 0.38
	48	EC_50_	2.94 ± 0.20

### Toxicity of fractions

**Fig. 1:**
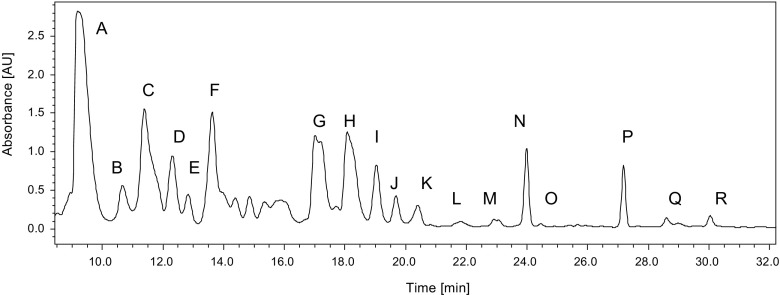
Chromatogram of extract obtained from *Woronichinia naegeliana* cells monitored at 220 nm. Letters match the fractions described in Table 2

**Fig. 2:**
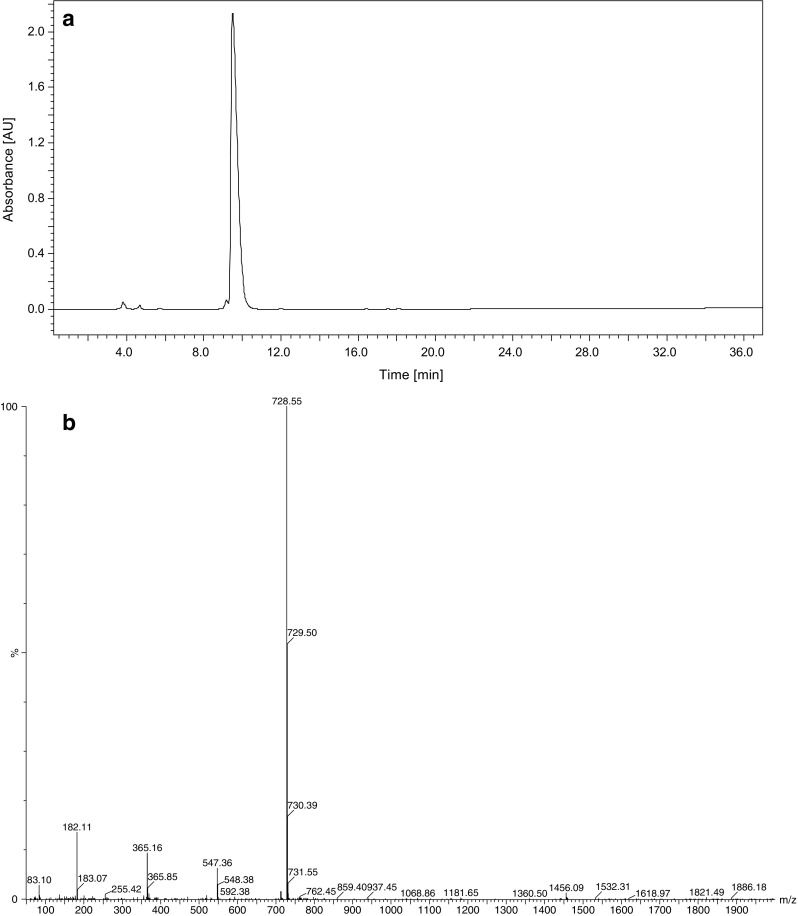

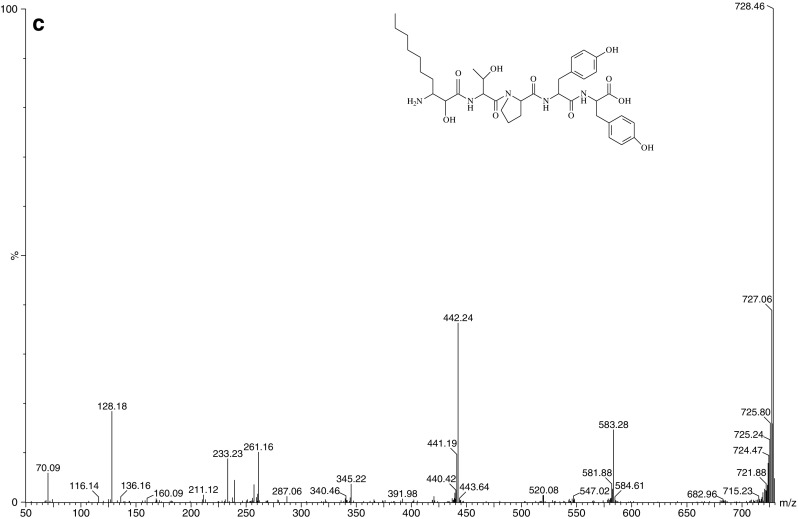
**a** Chromatogram of fraction A contained microginin FR3 monitored at 220 nm, **b** ion mass spectrum of microginin FR3 and **c** MS/MS product ion spectrum of microginin FR3 (m/z 728.5 Da) inside the structure of microginin FR3

The extract obtained from *W. naegeliana* cells was separated into 18 fractions (Fig. 1) that were used to determine which secondary metabolites are responsible for the toxicity of *W. naegeliana.* The bioactivity assays of separated fractions were based on the crustacean *T. platyurus* that was found to be more sensitive on cyanobacterial extract than *D. pulex*. One of the 18 fractions isolated from the cell extract exhibited biological activity in the tested concentration range, and the determined LC_50_ was equal to its content extracted from 5.34 mg d.w. mL^−1^ *W. naegeliana* cells. The remaining fractions containing secondary metabolites displayed a very low or no toxicity making it impossible to determine the LC_50_ value (Table 2). The bioactive fraction A (Fig. 2) contained pure microginin FR3 (MG-FR3) (purity 99%) for which determined LC_50_ was equal to 7.78 μg mL^−1^.

**Table 2:** Percent of dead *Thamnocephalus platyurus* larvae exposed for 24 h to separate fractions extracted from 10 mg d.w. mL^−1^ *Woronichinia naegeliana* cells. Data are expressed as mean ± SD (*n* = 5)

Fraction	Fraction composition^a^	Retention time (min)	(%) of dead *T. platyurus* larvae
A	Microginin FR3	9.2	100.0 ± 0.0
B	Microginin FR4	10.7	14.0 ± 5.5
C	Micropeptin T2	11.4	22.0 ± 8.4
D	Micropeptin 478-B	12.3	16.0 ± 11.4
E	Micropeptin 88D	12.8	30.0 ± 7.1
F	Cyanopeptolin 880	13.6	20.0 ± 0.0
G	Unknown compound 791 Da	17.0	24.0 ± 5.5
H	Microginin 757	18.1	16.0 ± 5.5
I	Cyanopeptolin 914, unknown compound 874 Da	19.0	6.0 ± 5.5
J	Unknown compounds—1013 and 973 Da	19.7	24.0 ± 8.9
K	Micropeptin SD999, unknown compound 1039 Da	20.4	22.0 ± 8.4
L	Unknown compound 1047 Da	21.8	32.0 ± 4.5
M	Microcystin-LR, microginin 91E, planktopeptin BL1061, unknown compound 1123 Da	23.1	26.0 ± 5.5
N	Cyanopeptolin 908, oscillamide B	24.0	10.0 ± 0.0
O	Cyanopeptolin B, unknown compound 888 Da	24.4	8.0 ± 4.5
P	Cyanopeptolin C, unknown compound 902 Da	27.2	40.0 ± 7.1
Q	Microginin 51A, cyanopeptolin D, unknown compounds—1061, 1021 and 812 Da	28.2	8.0 ± 4.5
R	Microginin 478	30.0	8.0 ± 4.5

^a^Fraction composition was determined according to Bober et al. 2011

### The enzyme inhibitory activity of MG-FR3

Protease assays including trypsin, chymotrypsin, elastase and thrombin were performed only for the MG-FR3, which gave positive result in bioassays with *T. platyurus*. The MG-FR3 did not affect the activity of tested enzymes (Table 3).

**Table 3:** The enzyme inhibitory activities of MG-FR3 (IC_50_—50% inhibitory concentration)

Enzyme	IC_50_ (μg mL^−1^)
Trypsin	>40
Chymotrypsin	>40
Elastase	>40
Thrombin	>100

## Discussion

Results showed different sensitivities of tested crustaceans to aqueous extract from *W. naegeliana* cells. A higher biological response to cyanobacterial extract was observed for *T. platyurus* rather than for *D. pulex* (Table 1). These results are consistent with the findings of Marsálek and Bláha (2004), who found that *T. platyurus* revealed the highest sensitivity to cyanobacterial samples among all tested commercially available biotests. A lower sensitivity to cyanobacterial extract was observed for *D. pulex*, although its toxicity increased with time. *Woronichinia naegeliana* has been described as a potential source of toxin by Scott (1991), but to the best of our knowledge, there are no published quantitative data relating to its toxicity. We compared our results with the values presented by Marsálek and Bláha (2004), who applied similar bioassays in the assessment of *Microcystis*-dominated water blooms. The determined toxicity of *W. naegeliana* cells was classified as average (Table 4). In studies presenting data and analyses of samples collected from blooms where *W. naegeliana* was the dominant species among secondary metabolites, different variants of microcystins have been detected (Willame et al. 2005, Baudin et al. 2006). Although a trace amount of microcystin-LR was also detected in the studied material, its toxicity values were higher than those obtained for *M. aeruginosa* cells containing a high amount of this hepatotoxin. Additionally, the neurotoxicity of samples collected from Finnish lakes was statistically associated with the presence of *Anabaena lemmermanni*, *Anabaena flos-aquae* and *W. naegeliana* cells in the samples (Sivonen et al. 1990). Neurotoxins were not detected in the studied *W. naegeliana* cells. However, the possibility that the extraction procedure used and the conditions of the liquid chromatography analyses were not optimal for the detection of the neurotoxins cannot be excluded. The divergences in these results might also be due to difficulties in the separation of particular cyanobacterial species from the collected samples.

**Table 4:** Comparison of the biological sensitivity of *Thamnocephalus platyurus* and *Daphnia pulex* exposed to aqueous extracts from various species of cyanobacteria. Data are presented as was described in Table 1

Cyanobacterial extract	*Thamnocephalus platyurus* 24-h LC_50_ (mg dry weight mL^−1^)	*Daphnia pulex* 24-h EC_50_ (mg dry weight mL^−1^)
*Woronichinia naegeliana* ^a^ (99%)^c^	0.99 ± 0.25	5.21 ± 0.38
*Microcystis aeruginosa* ^b^ (98%)^c^	0.11 ± 0.3	1.1 ± 1.2
*Microcystis ichtyoblabe* (75%)^c^ and *M. aeruginosa* ^b^ (20%)^c^	0.35 ± 0.1	2.1 ± 1.2
*Microcystis wesenbergii* ^b^ (98%)^c^	3.7 ± 1.9	12.3 ± 2.6

^a^Data obtained in this study

^b^Data published by Maršálek and Bláha (2004)

^c^Percent in parenthesis represents the content of a given species in the studied cyanobacterial biomass

In the majority of the toxicity tests conducted for the secondary metabolites produced by *W. naegeliana*, their biological impact on *T. platyurus* has not been determined. The exception is for microginin FR3 (Table 2). The difference between the LC_50_ of separated fractions and that obtained for the total *W. naegeliana* extract might be the result of the synergic effect of all the compounds. Most of the metabolites synthesized by *W. naegeliana* (Bober et al. 2011, 2014) belong to several classes of oligopeptides, which non-ribosomal synthesis is a significant part of cell metabolism (Welker and von Döhren 2006). Although their ecological role has not been fully elucidated, their inhibitory activity against proteases indicates that they are able to affect other organisms, even causing cell lysis. As a consequence, they might be a part of a protection strategy in the aquatic ecosystem (see references in Chlipala et al. 2011).

MG-FR3 belongs to the microginins, linear peptides that contain four to six amino acid residues with a characteristic N-terminal residue 3-amino-2-hydroxydecanoic acid (Ahda) and frequently two tyrosine units at the C-terminus (Welker and von Döhren 2006). Some of these oligopeptides have been recognized as leucine aminopeptidase (LAP) inhibitors due to the 2S configuration of the Ahda group (Ishida et al. 1997; Neumann et al. 1997). The structure N-Me-Tyr-Tyr at the C-terminus seemed to be crucial for the inhibition of angiotensin-converting enzyme (ACE) (Ishida et al. 2000). The inhibitory activity of some microginins for ACE was considered for the production of blood pressure-decreasing drugs (Kraft et al. 2006). However, some microginins did not show any inhibition activity against trypsin, chymotrypsin, papain, elastase or protein phosphatase 1A (Ishida et al. 1997).

MG-FR3, which was detected in the extract from *W. naegeliana* cells, induced a biological response in crustaceans and therefore was used in assays including digestive proteases such as trypsin, chymotrypsin and elastase. The results are consistent with reports of other microginins showing that MG-FR3 did not affect the activity of tested enzymes (Table 3). No inhibition of thrombin was observed. Therefore, the observed toxicity of *W. naegeliana* towards planktonic invertebrates seems to be connected to a mode of action other than serine protease inhibition or may be a result of the synergic effect of all the produced cyanopeptides. To date, the significance of these oligopeptides to cyanobacteria metabolism and their biological function is unclear. There are several hypotheses concerning cyanopeptides, including the mechanism of the inhibition of vital enzymes, allelopathic activity on other phytoplankton organisms, chemical grazing protection and involvement in cell metabolism (see references in Halstvedt et al. 2008). However, the modes of action require further investigation.

In conclusion, the biological activity of the poorly studied freshwater bloom forming cyanobacteria *W. naegeliana* towards planktonic invertebrates was studied. The biological effects on planktonic crustaceans were related to a fraction containing MG-FR3. Effective assessment of the environmental risk connected to the occurrence of cyanobacterial blooms should also take into account others than those commonly known to produce toxic secondary metabolites.
